# UV-B-Induced Inflammasome Activation Can Be Prevented by Cis-Urocanic Acid in Human Corneal Epithelial Cells

**DOI:** 10.1167/iovs.61.4.7

**Published:** 2020-04-09

**Authors:** Eveliina Korhonen, Jovana Bisevac, Juha M. T. Hyttinen, Niina Piippo, Maria Hytti, Kai Kaarniranta, Goran Petrovski, Anu Kauppinen

**Affiliations:** 1 School of Pharmacy, Faculty of Health Sciences, University of Eastern Finland, Kuopio, Finland; 2 Department of Clinical Chemistry, HUSLAB, Helsinki University Hospital, Helsinki, Finland; 3 Center for Eye Research, Department of Ophthalmology, Oslo University Hospital, Oslo, Norway; 4 Institute of Clinical Medicine, University of Oslo, Oslo, Norway; 5 Department of Ophthalmology, Institute of Clinical Medicine, University of Eastern Finland, Kuopio, Finland; 6 Department of Ophthalmology, Kuopio University Hospital, Kuopio, Finland

**Keywords:** ultraviolet B, inflammasome, urocanic acid, cornea, inflammation

## Abstract

**Purpose:**

The cornea is continually exposed to highly energetic solar UV-B (280-320 nm). Our aim was to investigate whether UV-B triggers the activation of NLRP3 inflammasomes and the production of IL-1β and/or IL-18 in human corneal epithelial (HCE) cells. Additionally, we studied the capability of cis-urocanic acid (cis-UCA) to prevent inflammasome activation or alleviate inflammation through other signaling pathways.

**Methods:**

HCE-2 cell line and primary HCE cells were primed using lipopolysaccharide or TNF-α. Thereafter, cells were exposed to UV-B before or after the addition of cis-UCA or caspase-1 inhibitor. Caspase-1 activity was measured from cell lysates by an enzymatic assay. IL-1β, IL-18, IL-6, IL-8, and NLRP3 levels were detected using the ELISA method from cell culture media. Additionally, intracellular NLRP3 levels were determined by the Western blot technique, and cytotoxicity was measured by the LDH assay.

**Results:**

UV-B exposure significantly increased caspase-1 activity in TNF-α-primed HCE cells. This result was consistent with the concurrently induced IL-1β secretion. Both caspase-1 activity and release of IL-1β were reduced by cis-UCA. Additionally, UV-B stimulated the caspase-1-independent production of IL-18, an effect also reduced by cis-UCA. Cis-UCA decreased the release of IL-6, IL-8, and LDH in a time-dependent manner when administered to HCE-2 cells after UV-B exposure.

**Conclusions:**

Our findings demonstrate that UV-B activates inflammasomes in HCE cells. Cis-UCA can prevent the secretion of IL-1β and IL-18 and therapeutically reduces the levels of IL-6, IL-8, and LDH in UV-B-stressed HCE cells.

The cornea absorbs a significant amount of ambient UV-B (290-320 nm) and protects the deeper ocular structures from harmful radiation.^1^^–^^3^ Although UV-B accounts for only 4% of terrestrial light, it is light with a highly energetic wavelength, causing many harmful eye conditions, such as painful photokeratitis and climatic droplet keratopathy.^34^ At the cellular level, excessive UV-B evokes corneal phototoxicity including direct DNA damage, such as the formation of cyclobutane pyrimidine dimers^5^ and disturbances in the antioxidant balance resulting in cellular oxidative stress.^6^^–^^8^

IL-1β and IL-18 are pro-inflammatory cytokines that have a major role in inflammatory responses.^9^^,^^10^ The secretion of these cytokines is regulated by intracellular multiprotein platforms called inflammasomes. Nucleotide-binding domain and leucine-rich repeat pyrin-containing protein 3 (NLRP3) is the receptor of the most extensively studied inflammasome^11^^,^^12^; it can be activated in response to nonsterile (microbes) or sterile stressors.^11^ NLRP3 activation has to be preceded by a priming step,^11^^,^^12^ which triggers the translation of NLRP3 and pro-IL-1β via nuclear factor-kappa B (NF-κB) signaling.^13^ IL-18 precursor protein is constitutively expressed in most of human cells (e.g., in the corneal epithelial cells, peripheral blood mononuclear cells, keratinocytes, endothelial cells, and retinal pigment epithelial cells).^14^^–^^17^

Inflammasome activation occurs when primed cells sense pathogen-associated molecular patterns or danger-associated molecular patterns via the NLRP3, which leads to NLRP3 oligomerization and the assembly of the inflammasome multiprotein complex also containing the adaptor protein apoptosis-associated speck-like protein containing a C-terminal caspase recruitment domain (ASC) and the cysteine protease procaspase-1.^18^^,^^19^ The formation of the inflammasome complex causes the proteolytic activation of caspase-1,^20^^,^^21^ which enables the cleavage of pro-IL-1β into its biologically active and secreted form.^21^ In conjunction with the cleavage of pro-IL-1β, pro-IL-18 requires proteolytic processing before secretion.^22^

Urocanic acid (UCA) is a naturally occurring endogenous chromophore produced from histidine in the upper layer of the epidermis in a deamination reaction catalyzed by histidase.^23^ UCA usually occurs as a *trans* form, but is converted to the *cis* isomer in a photoisomerization reaction by UV-B.^23^^,^^24^ Therapeutic effects of cis-UCA have been studied for nearly three decades. Despite the fact that the effects of cis-UCA may be cell type-dependent, many *in vivo* and *in vitro* studies have revealed its anti-inflammatory properties and potential to protect from cell injury.^25^^–^^28^ Our previous studies have revealed that cis-UCA is well tolerated by human corneal epithelial (HCE) and conjunctival epithelial (HCEC) cells.^29^ Additionally, pretreatment with cis-UCA prevents cell death and the secretion of IL-8 and IL-6 in UV-B-induced HCE and HCEC cells.^29^ Moreover, we have shown that cis-UCA could reduce the activation of activator protein-1 and mitogen-activated protein kinase pathways in the UV-B-irradiated HCE-2 cell line.^30^

Although it is evident that excessive UV-B exposure can induce an acute inflammatory response in the cornea, its role in inflammasome signaling is unknown. Because inflammasomes are key players in inflammation, we have now investigated whether the UV-B-induced inflammation is regulated by the inflammasomes in HCE cells and whether it can be prevented by cis-UCA. We have also explored the therapeutic potential of cis-UCA by investigating whether it reduces IL-6, IL-8, or LDH release when administered after UV-B exposure.

## Materials and Methods

### Cell Stimulations

The human corneal epithelial cell line (HCE-2) was purchased from the American Type Culture Collection. HCE-2 cells were cultured in the Keratinocyte Serum Free growth medium (Life Technologies, Paisley, UK) containing 50 µg/mL bovine pituitary extract, 5 ng/mL human recombinant epidermal growth factor 1-53 (EGF 1-53; both from Life Technologies, Grand Island, NY, USA), 100 U/mL penicillin (Lonza, Walkersville, MD, USA), 100 µg/mL streptomycin (Lonza), and 0.005 mg/mL insulin (Sigma Aldrich, Saint Louis, MO, USA) at 37°C in a humidified atmosphere with 5% CO_2_. Cells in passage numbers ranging from 69 to 86 were used in the study. HCE cell culture plates for the maintenance (100 mm x 20 mm; Sigma Aldrich, St. Louis, MO, USA) and 12-well culture plates for the experiments (Corning Inc., Corning, NY, USA) were coated with 0.01 mg/mL fibronectin (Sigma-Aldrich), 0.03 mg/mL collagen (STEMCELL technologies, Vancouver, Canada), and 0.01 mg/mL bovine serum albumin (Roche Diagnostics GmbH, Mannheim, Germany) in the Keratinocyte Serum Free Medium. The coating solution was incubated for 30 to 90 minutes at 37°C until replaced by cell suspensions. In the experiments, cells were seeded on 12-well plates at a density of 1.5 × 10^5^ cells/mL and incubated for 24 hours in a humidified 5% CO_2_ incubator at 37°C.

All tissue collections complied with the Guidelines of the Helsinki Declaration and were approved by the Local Ethical Committees (No 2017/418). Human limbal biopsies, obtained from cadaveric corneo-scleral rings after corneal transplantation were treated with Dispase II (2.4 U/mL, Roche Diagnostics) for 10 minutes at 37°C and thereafter blocked with fetal bovine serum (Sigma-Aldrich), plated on six-well plates (Corning Inc.) with the epithelial side down and covered with cell culture medium. Once the limbal biopsies were attached, they were completely covered and maintained in DMEM/F12 medium (Thermo Fisher Scientific, Waltham, MA, USA) containing 100 U/mL penicillin, 100 µg/mL streptomycin, 1.25 µg/mL amphotericin B, 5% FBS, 2 ng/mL human epidermal growth factor, 5 µg/mL insulin-transferrin-sodium selenite, 15 µM hydrocortisone, 0.5% dimethylsulfoxide, and 30 ng/mL cholera toxin A (all from Sigma Aldrich) in a humidified 5% CO_2_ incubator at 37°C. After the primary HCE (pHCE) cells growing out from the limbal biopsies had reached confluency, they were treated with 0.25% Trypsin-EDTA (Sigma Aldrich) and seeded on 12-well plates (Corning Inc.) at a density of 2.5 × 10^5^ cells/mL and incubated for 48 to 72 hours in a humidified 5% CO_2_ incubator at 37°C. Thereafter, the cell layers were washed and replaced with DMEM/F12 medium (Thermo) without any supplements.

The priming signal for pHCE and HCE-2 cells was provided by tumor necrosis factor α (TNF-α, 10 ng/mL; R&D Systems, Minneapolis, MN, USA) or lipopolysaccharide (LPS, 1 µg/mL; Sigma Aldrich) by incubating cells for 24 h in a humidified 5% CO_2_ incubator at 37°C. Thereafter, cell cultures were irradiated with UV-B (two TL 20W/12 tubes, Philips, Eindhoven, The Netherlands) at the energy level of 0.2 J/cm^2^. The UV-B lamps were calibrated using a spectroradiometer (Waldman Variocontrol, Germany) by Istekki Ltd. (Kuopio, Finland) before the experiments. The irreversible caspase-1 inhibitor, Ac-YVAD-CMK (200 µM for HCE-2 cells; Calbiochem, San Diego, CA, USA; or 100 µM for pHCE cells; Sigma Aldrich) was added to the wells 1 hour before the UV-B irradiation, where applicable. Additionally, cells were treated with cis-urocanic acid (cis-UCA, 100 µg/mL, Sigma Aldrich) 1 hour before or immediately after the UV-B exposure. The concentration of cis-UCA had been optimized in our previous study.^29^

### Sample Collection

Cell culture medium samples and whole cell lysates were collected 0.5 to 48 hours after the exposure to UV-B or cis-UCA, depending on the experiment. Cell culture medium samples were centrifuged at 380 × *g* for 10 minutes and supernatants were transferred into clean tubes. The cells were rinsed with ice-cold 1x Dulbecco's phosphate-buffered saline (Sigma Aldrich) and lysed by addition of the M-PER solution (Thermo Fisher Scientific) or the cell lysing buffer provided by the manufacturer of the caspase-1 activity kit (R&D). Cell lysates prepared using the M-PER solution were centrifuged at 16 000 × *g* for 10 minutes at 4°C. In the measurements of caspase-1 activity, cells from two to six nonirradiated or four to 12 UV-B-irradiated parallel wells were collected into one tube and centrifuged according to the manufacturers protocol. Before the analyses, protein concentrations were measured using the Bradford method.^31^

### ELISA Measurements

IL-1β, IL-6, IL-8 (BD Biosciences, San Diego, CA, USA), IL-18 (eBioscience, San Diego, CA, USA), and NLRP3 (Cusabio, Wuhan, China) were measured using the ELISA method from the cell culture medium according to the manufacturer's instructions. Absorbance values were measured at 450 nm with the reference wavelength of 655 nm by a spectrophotometer (Biorad, Model 550, Hercules, CA, USA).

### Caspase-1 Activity

Caspase-1 activity was measured using the caspase-1 colorimetric assay according to the manufacturer's protocol. Absorbance values were determined at a wavelength of 405 nm by a spectrophotometer (ELx808, Biotek Instruments Inc., Winooski, VT, USA). The kit supplements without cell lysates were used as blank samples. Thereafter, blank-subtracted results were normalized to protein concentrations and expressed as a ratio to the untreated cells.

### Cytotoxicity Measurements

The quantity of LDH was measured from cell culture medium samples using the CytoTox96 Non-Radioactive Cytotoxicity assay (Promega Corporation, Madison, WI, USA) according to the manufacturer's instructions. Colorimetric reactions were monitored at a wavelength of 490 nm in a spectrophotometer (Biorad).

### Western Blot

Equal amounts of protein (20-40 µg in HCE-2 cell lysates or 5-10 µg in primary HCE cell lysates) were run in 10% SDS-PAGE gels and wet-blotted overnight onto nitrocellulose membranes (GE Healthcare, Little Chalfont, Buckinghamshire, UK). Ponceau S (Sigma-Aldrich) staining was used to ensure the accurate protein transfer from the gels to the membranes. The membranes were blocked with 5% milk in 0.1% Tween-20/Tris-buffered saline for 1 hour at room temperature (RT). The Tween/Tris-buffered saline solution was also used as a washing buffer and a diluent for the antibodies. Thereafter, the membranes were incubated with an anti-CIAS1/NALP3 (1:1000; cat. 109314, Abcam, Cambridge, UK) monoclonal rabbit antibody overnight at +4°C. The membranes were washed 3 × 5 minutes with washing buffer followed by a 2-hour incubation at RT with a horseradish peroxidase-conjugated anti-rabbit IgG antibody (1:5 000, cat. A16104, Novex) in blocking buffer. The washing steps were repeated and the NLRP3 protein-antibody complexes were detected using the enhanced chemiluminescent assay after the addition of the substrate (Millipore, Billerica, MA, USA). The band intensities given by NLRP3 were normalized to α-tubulin values using a monoclonal mouse α-tubulin (1:8000; cat. T5168, Sigma Aldrich). Following the incubation for 15 minutes at RT, membranes were washed three times for 5 minutes. Polyclonal horseradish peroxidase-conjugated sheep anti-mouse IgG (1:10 000; cat. NA931, GE Healthcare) was used as a secondary antibody and incubated for 15 minutes at RT before performing the washing and detection steps as described previously. All the results were quantified using the ImageJ program (*http://rsb.info.nih.gov/ij/index.html*).

### Confocal Imaging of DsRed-ASC

HCE-2 cells were cultured on eight-well chamber slides (Ibidi GmbH, Martinsried, Germany) at a density of 4.5 × 10^4^ cells/well. Sub-confluent (70%-80%) cell layers were transfected with DsRed-ASC plasmid construct (20 ng) along with ExGen500 transfection reagent (Thermo Fischer Scientific, Waltham, MA, USA). The cells were incubated for 24 hours in a humidified 5% CO_2_ incubator at 37°C followed by a priming with TNF-α (10 ng/mL) for the next 24 hours. Thereafter, ASC specks were observed under the confocal microscope (Zeiss Axio Observer inverted microscope with Zeiss LSM 700 confocal module) at 5 and 24 hours upon UV-B exposure (0.2 J/cm^2^).

### Statistical Analyses

All results were analyzed using the GraphPad Prism program (GraphPad Software Version 7.04, San Diego, CA, USA). Pairwise comparisons were performed using the Mann-Whitney *U*-test, and *P* values ≤ 0.05 were considered statistically significant.

## Results

### UV-B Exposure Induces Caspase-1 Dependent Secretion of IL-1β in HCE Cells

**Figure 1.**
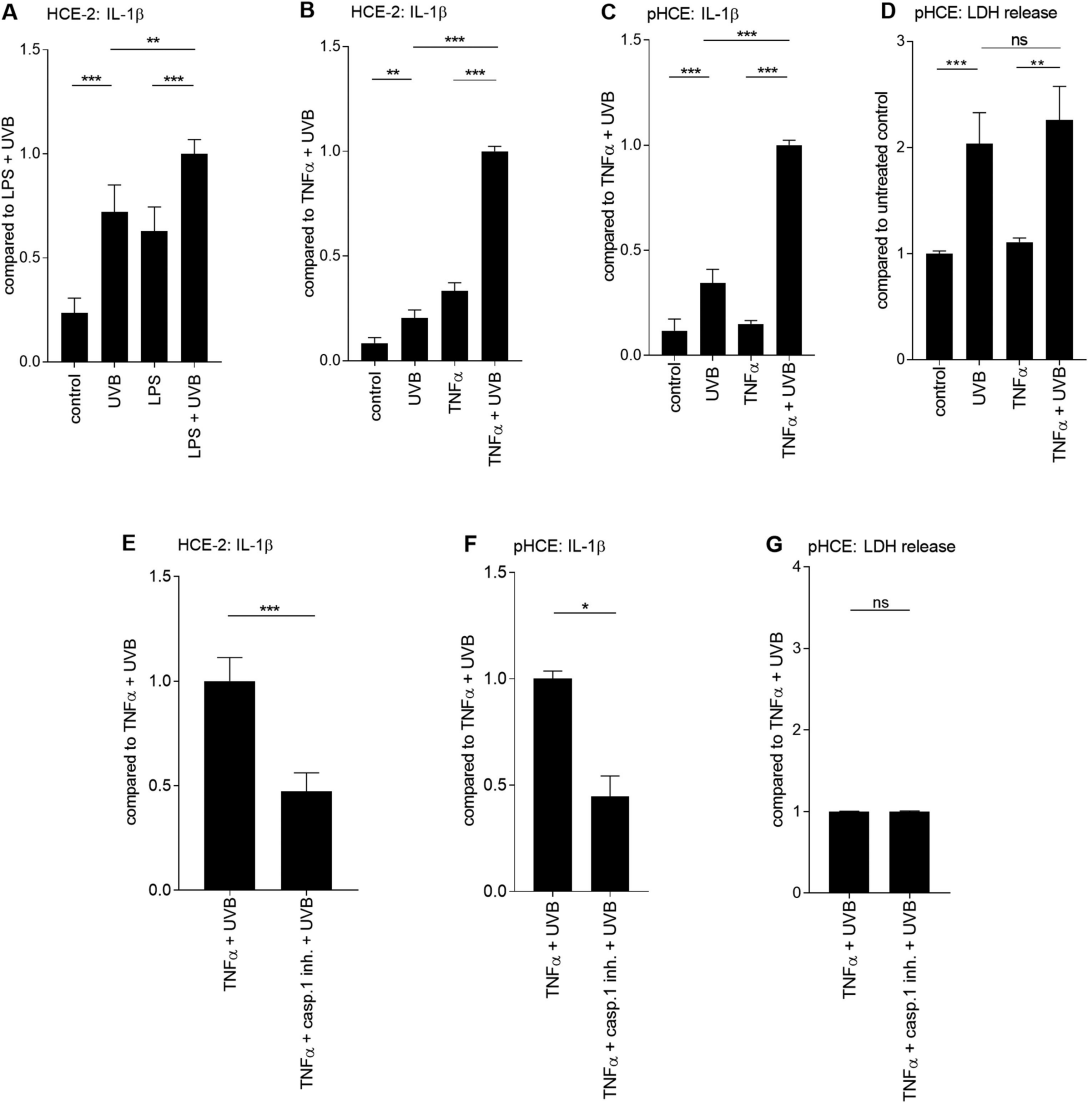
**Caspase-1-dependent secretion of IL-1β in** **UV-B-stimulated HCE-2 and primary HCE cells.** HCE-2 cells were primed for 24 hours either with LPS (1 µg/mL; A) or TNF-α (10 ng/mL; B) followed by irradiation with UV-B (0.2 J/cm^2^). The secretion of IL-1β was measured by the ELISA method from HCE cell culture media for 24 hours after the UV-B irradiation. The levels of IL-1β were combined from three independent experiments with five to six parallel samples per group. Additionally, levels of IL-1β and LDH were measured from the primary HCE cell culture media from eight donors with one to three parallel samples per donor (C-D). Alternatively, HCE-2 (E) or primary HCE (F-G) cells were pretreated with the caspase-1 inhibitor (200 µM [E] or 100 µM [F-G] for 1 hour before the UV-B exposure). IL-1β data from HCE-2 cells (E) have been combined from three independent experiments each with 10 to 12 parallel samples. IL-1β and LDH data from cultured primary HCE cells (F-G) have been combined from four donors with one to two parallel samples per donor. All data are presented as mean ± SEM. *P < 0.05, **P < 0.01, ***P < 0.001, ns = not significant, Mann-Whitney *U*-test; pHCE = primary HCE cells; casp.1 inh. = caspase-1 inhibitor.

To examine the ability of UV-B to activate inflammasomes in HCE-2 cells, cell cultures were primed with LPS or TNF-α and subsequently exposed to UV-B light. LPS and TNF-α-primed and UV-B-treated HCE-2 cells secreted higher amounts of IL-1β (1.6-fold in the LPS-treated cells and 3.0-fold in the TNF-α-primed cells) compared with LPS or TNF-α-treated controls (mean for LPS: 0.3 pg/ml vs. LPS + UV-B: 0.4 pg/mL, *P* < 0.001, Fig. 1 A; TNF-α: 0.5 pg/mL vs. TNF-α + UV-B: 1.7 pg/mL, *P* < 0.0001, Fig. 1 B). Additionally, UV-B without priming tripled the IL-1β levels when compared to untreated control cells (mean for untreated control: 0.1 pg/mL vs. UV-B: 0.3 pg/mL; Figs. 1 A–B) although the levels were 1.4 (*P* < 0.01) and 4.1 (*P* < 0.0001) times lower when compared to LPS or TNF-α-primed and UV-B-treated cells, respectively.

The results with the pHCEs were in line with those obtained with HCE-2 cells, showing that the secretion of IL-1β was increased after the UV-B exposure (untreated control: 22.2 pg/mL vs. UV-B: 111.0 pg/mL, *P* < 0.0001; TNF-α: 52.0 pg/mL vs. TNF-α + UV-B: 326.2 pg/mL, *P* < 0.0001; Fig. 1 C). Once again, TNF-α + UV-B-treated cells significantly augmented the production of IL-1β when compared with UV-B-treated cells without priming (*P* < 0.0001; Fig. 1 C). Overall, TNF-α + UV-B-treatment induced a much greater, (i.e., 190-fold elevations) response of IL-1β in pHCE cells (TNF-α + UV-B: 326.2 pg/mL) in comparison to the HCE-2 cell line (TNF-α + UV-B: 1.7 pg/mL). LDH data showed that the increased cytokine levels were not attributable to the passive leakage of proteins (UV-B vs. TNF-α + UV-B, *P* > 0.05; Fig. 1 D). Moreover, the addition of a selective caspase-1 inhibitor significantly mitigated the secretion of IL-1β in UV-B-irradiated HCE-2 (*P* < 0.001, Fig. 1 E) and cultured primary HCE cells (*P* < 0.05; Fig. 1 F), evidence of a role in inflammasome signaling in the production of IL-1β. It was confirmed with the LDH assay that caspase-1 inhibitor was not toxic to the HCE cells (TNF-α + UV-B vs. TNF-α + caspase-1 inhibitor + UV-B, *P* > 0.05; Fig. 1 G).

### UV-B Induces the Caspase-1-Independent Secretion of IL-18 in Human Corneal Epithelial Cells

**Figure 2.**
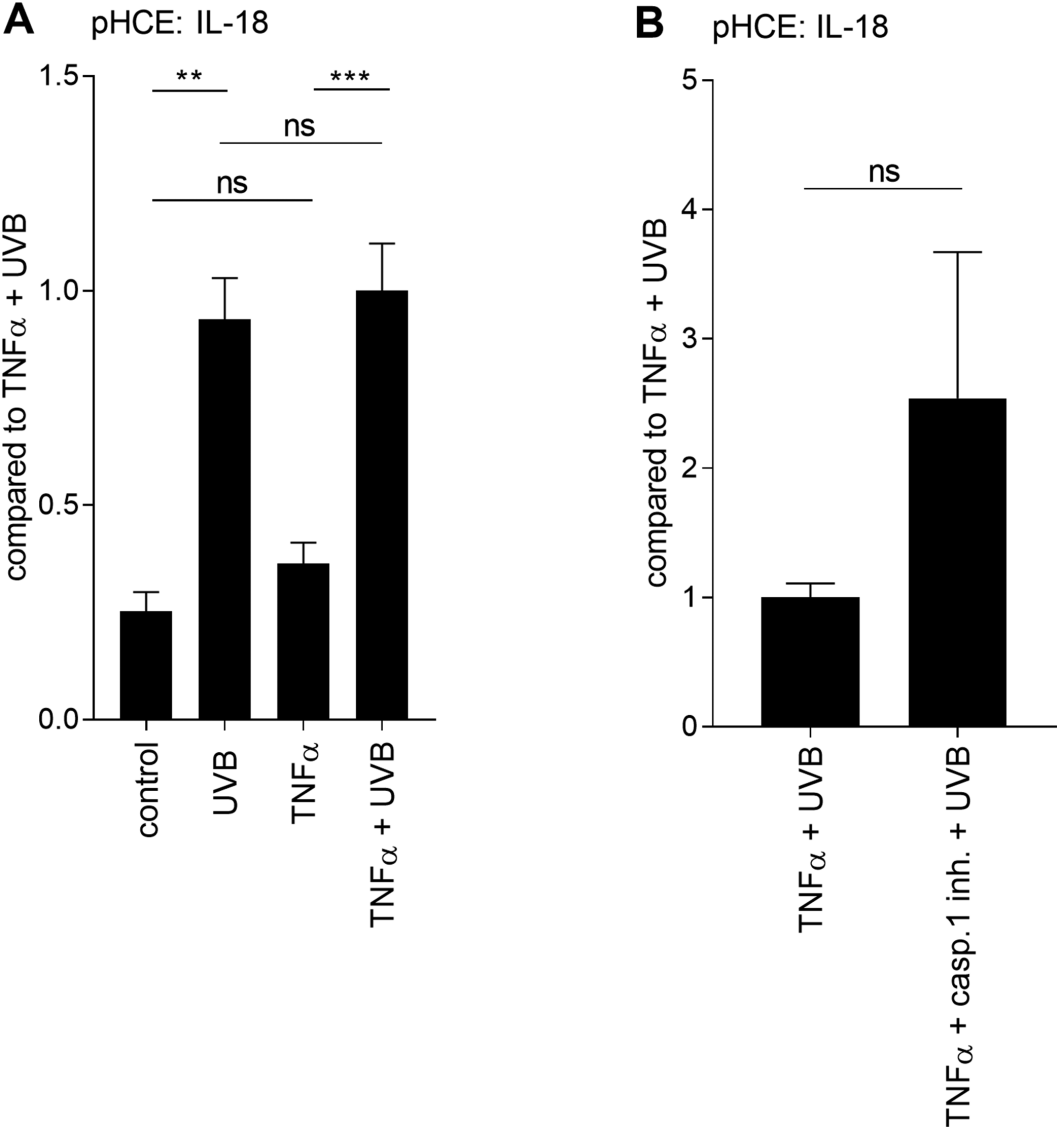
**Caspase-1-independent release of IL-18 in** **UV-B-irradiated primary HCE cells.** Primary HCE cells were treated with TNF-α (10 ng/mL) for 24 hours followed by irradiation under UV-B light (0.2 J/cm^2^). The secretion of IL-18 was measured for 24 hours after the UV-B exposure from the cell culture medium samples (A). Alternatively, cells were pretreated with the caspase-1 inhibitor (100 µM) at 1 hour before UV-B irradiation (B). ELISA results of extracellular IL-18 have been combined from (A) three or (B) two donors with two parallel samples per donor. Data are represented as mean ± SEM. **P < 0.01, ***P < 0.001, ns = not significant, Mann-Whitney *U*-test; casp.1 inh. = caspase-1 inhibitor.

Next, we evaluated whether the secretion of IL-18 was upregulated after the UV-B exposure in HCE cells. Instead of LPS priming, only TNF-α was used to prime cells since it resulted in 4.25 times higher concentration of IL-1β in UV-B-irradiated HCE-2 cells (LPS + UV-B: 0.4 pg/mL and TNF-α + UV-B: 1.7 pg/mL; Figs. 1 A-B). In line with the IL-1β findings, the secretion of IL-18 was increased after the UV-B irradiation (untreated control: 23.7 pg/mL, UV-B: 98.1 pg/mL; *P* < 0.01). Similarly, UV-B-induced the secretion of IL-18 in TNF-α-treated pHCE cells (TNF-α: 40.0 pg/mL, TNF-α + UV-B: 105.3 pg/mL; *P* < 0.001; Fig. 2 A). There was no difference in the levels of IL-18 when UV-B and TNF-α + UV-B-treated cells were compared with each other (*P* > 0.05; Fig. 2 A). Surprisingly, treatment with the caspase-1 inhibitor did not reduce the secretion of IL-18 (*P* > 0.05; Fig. 2 B), suggesting that UV-B does not cause the maturation IL-18 via a caspase-1-dependent mechanism in HCE cells.

### NLRP3 is Secreted After the UV-B Irradiation from TNF-α-Primed Human Corneal Epithelial Cells

**Figure 3.**
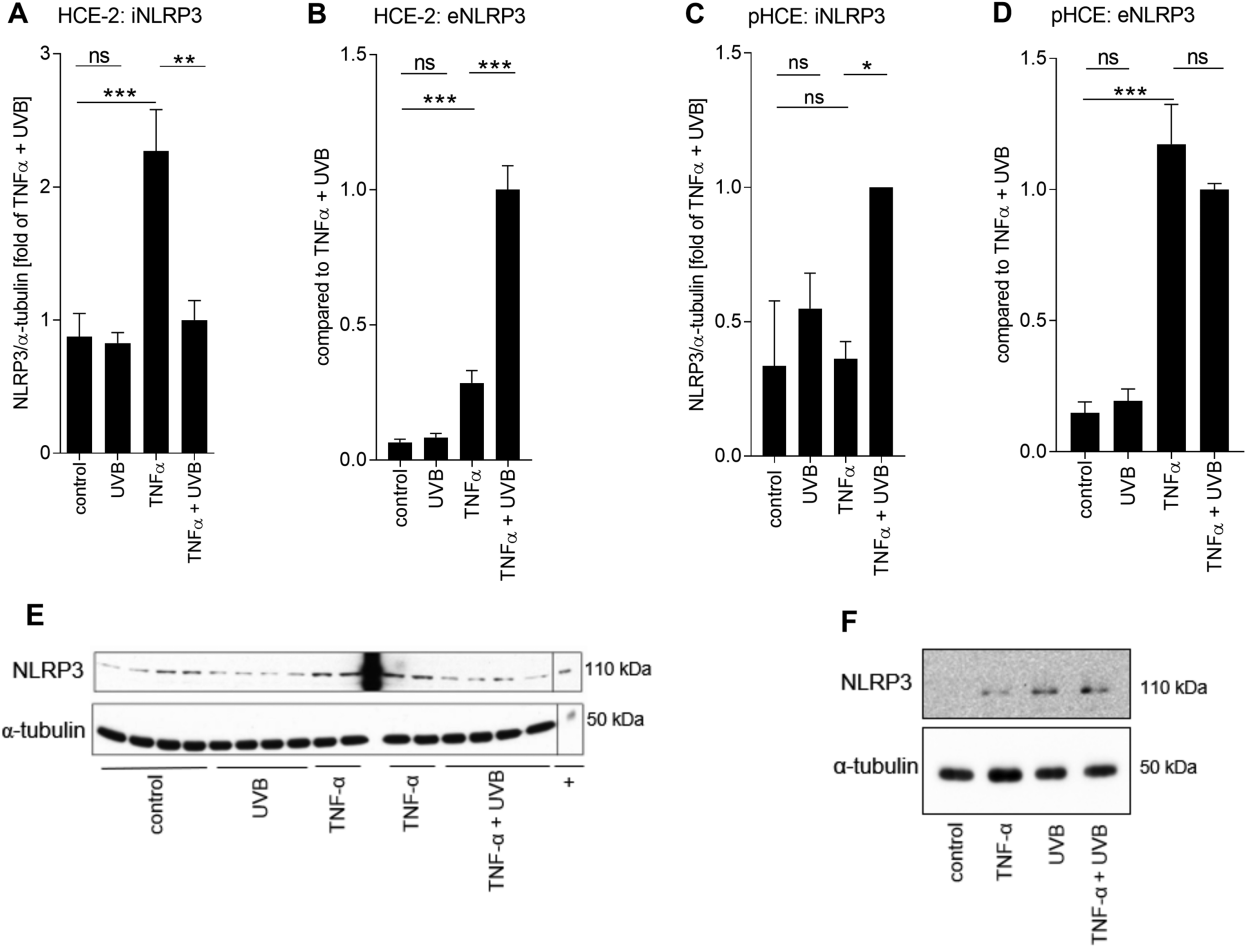
**Fate of NLRP3 in** **UV-B-stressed HCE cells.** HCE-2 cells were primed for 24 hours with TNF-α (10 ng/mL) following irradiation with UV-B (0.2 J/cm^2^), and then incubated for 24 hours before sample collection and analysis. Intracellular levels of NLRP3 (iNLRP3; A) were measured by the Western blot technique from cell lysates and the levels of NLRP3 were normalized to those of α-tubulin. Data in panel A is combined from two (UV-B) to four (untreated control, TNF-α, TNF-α + UV-B the correct group is TNF-α + UVB. This means that extra red comma should be removed.) independent experiments with three to four parallel samples in each group. Extracellular levels of NLRP3 (eNLRP3; B) were measured by the ELISA method from HCE-2 cell culture supernatants and data were combined from three independent experiments with three parallel samples in each group. Experiments were repeated using primary HCE cells, and intracellular levels of NLRP3 were measured by Western blot from cells of four donors (C), and extracellular levels of NLRP3 by ELISA from four donors with two parallel samples (D). (E, F) Representative Western blots from NLRP3 and α-tubulin measurements of (A) HCE-2 cells lysates or (C) primary HCE cell lysates. All data are presented as mean ± SEM. *P < 0.05, **P < 0.01, ***P < 0.001, ns = not significant, Mann-Whitney *U*-test; + the NLRP3 positive control + equals to the NLRP3 positive control.

TNF-α significantly increased the levels of intracellular NLRP3 in HCE-2 cells (*P* < 0.001; Fig. 3 A), suggesting an efficient priming signal for the cells. NLRP3 was released out of the cells following the inflammasome activation, as shown by the 2.3-fold reduction in the levels of intracellular NLRP3 (*P* < 0.01) and the 3.5-fold increase in the cell culture medium (*P* < 0.001) in TNF-α + UV-B-treated HCE-2 cells in comparison to TNF-α-treated control cells (Figs. 3 A-B). Additionally, TNF-α induced the secretion of NLRP3 in comparison to untreated controls (*P* < 0.001; Fig. 3 B), though the levels were 3.5 times lower when compared with the TNF-α + UV-B group. Exposure to UV-B without the priming signal was not essential for the induction of NLRP3 in HCE-2 cells since the intracellular or extracellular levels of NLRP3 showed no change upon exposure (*P* > 0.05,Figs. 3 A-B). Unlike HCE-2 cells, intracellular NLRP3 levels were increased in TNF-α-primed pHCE cells after the UV-B exposure (*P* < 0.05; Fig. 3 C). Moreover, NLRP3 was released out of the primed pHCE cells after the UV-B exposure, while instead of UV-B (*P* > 0.05; Fig. 3 D), TNF-α highly induced the secretion of NLRP3 (*P* < 0.001; Fig. 3 D) when compared with untreated control.

### UV-B Affects Formation of ASC Specks in Human Corneal Epithelial Cells

**Figure 4.**
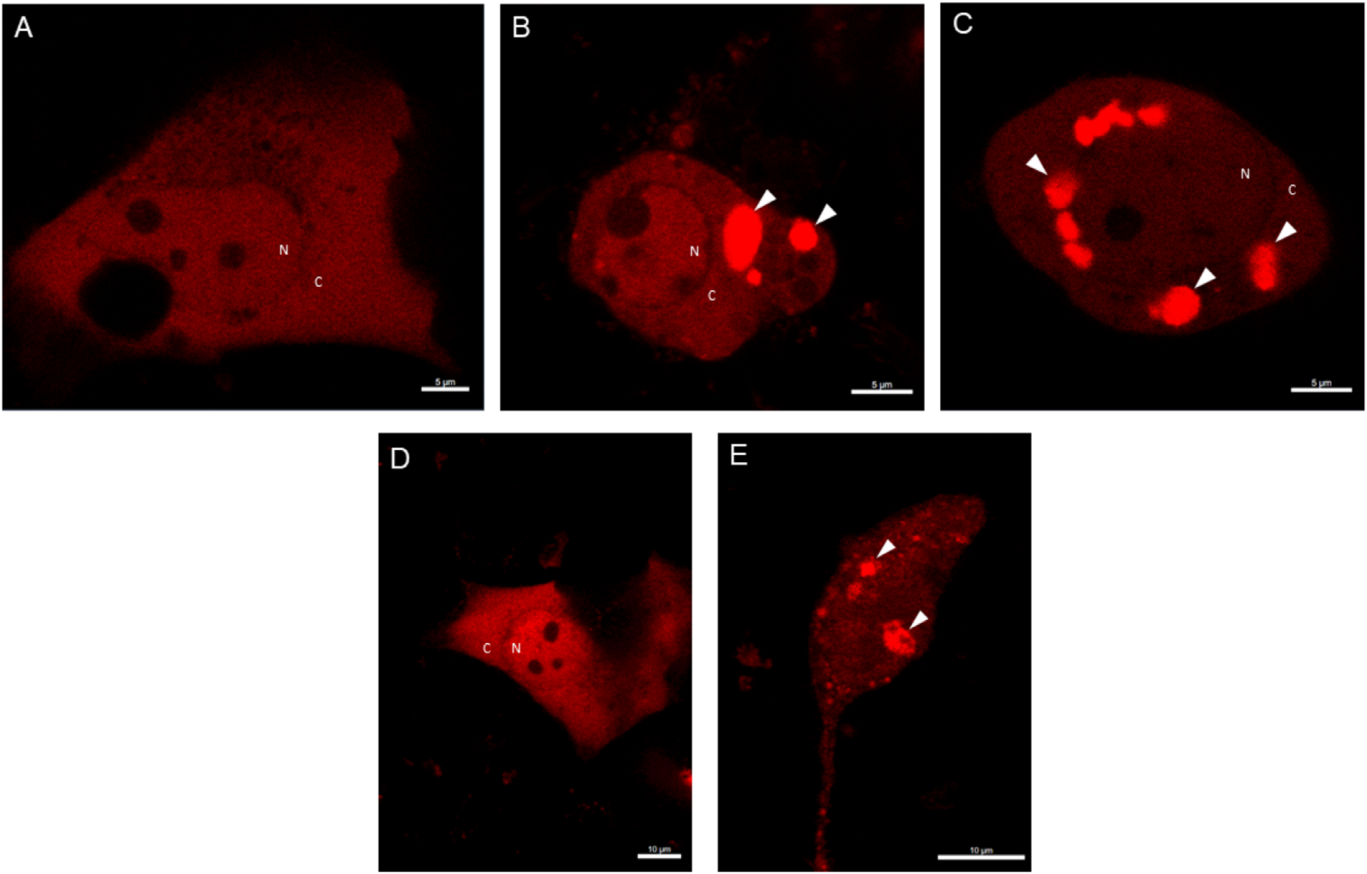
**The formation of ASC specks upon** **UV-B** **irradiation in HCE cells.** HCE-2 cells were transfected with DsRed-ASC plasmid constructs for 24 hours and primed for the next 24 hours with TNF-α (10 ng/mL). HCE-2 cells were observed under the confocal microscope 5 hours after the UV-B exposure (0.2 J/cm^2^; A = control group and B-C = TNF-α + UV-B treated group). Alternatively, microscopic examinations were performed 24 hours after the UV-B irradiation (D = control group and E = TNF-α + UV-B treated group). Experiments were repeated three times and images were photographed using the 63-fold objective (A-C: scale bar, 5 µm; D-E: scale bar, 10 µm). White arrows indicate ASC specks. N = nucleus, C = cytoplasm.

Thereafter, we studied further the activation of NLRP3 by measuring ASC specks upon UV-B irradiation in HCE-2 cells under the confocal microscope. UV-B clearly induced the formation of ASC specks already 5 hours upon UV-B exposure when compared with untreated control (Figs. 4 A-C). Additionally, ASC specks were observed at the 24 h timepoint upon UV-B irradiation in TNF-α + UV-B-irradiated cells but not in untreated control (Figs. 4 D-E). These findings support our other data that UV-B exposure in primed HCE cells activates the assembly of inflammasomes.

### cis-UCA Prevents the UV-B-Induced Secretion of IL-1β, IL-18, and LDH from Human Corneal Epithelial Cells

**Figure 5.**
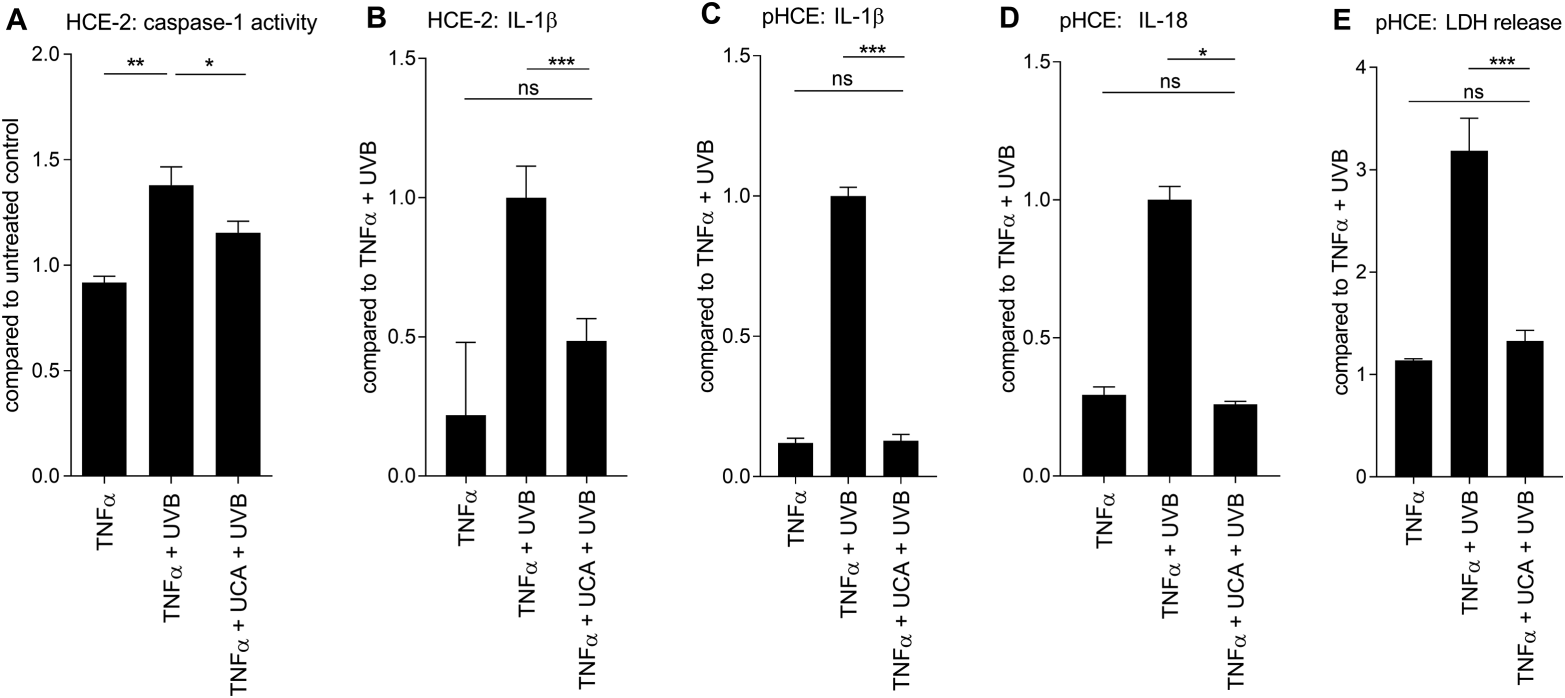
**Preventive effect of cis-UCA on the secretion of IL-1β and IL-18 in** **UV-B-stressed HCE cells.** TNF-α–primed (10 ng/mL) HCE cells were pretreated with cis-UCA (100 µg/mL) for 1 hour followed by irradiation with UV-B (0.2 J/cm^2^). (A) Caspase-1 activity was measured from HCE-2 cell lysates by an enzymatic assay and (B) IL-1β levels from HCE-2 cell culture medium samples by the ELISA method at 24 hours after the UV-B exposure. Data for caspase-1 activity were combined from two independent experiments with three parallel samples per group, and data of IL-1β were combined from three independent experiments with 10 to 12 parallel samples per group in each experiment. The results were confirmed in primary HCE cells where the amounts of (C) IL-1β, (D) IL-18, and (E) LDH were measured. Levels of IL-1β (C) and LDH (E) were investigated from four donors with three (TNF-α) or two (UV-B, TNF-α + UV-B, TNF-α + cis-UCA + UV-B) parallel samples from each donor. (D) IL-18 was measured from two donors with two parallel samples from each donor. All data are shown as mean ± SEM. *P < 0.05, **P < 0.01, ***P < 0.001, ns = not significant, Mann-Whitney *U*-test.

Next, the ability of cis-UCA to modulate the UV-B-induced inflammasome activation was studied. Cis-UCA reduced the UV-B-induced caspase-1 activity in HCE-2 cells (*P* < 0.05; Fig. 5 A). Subsequent experiments with the HCE-2 cell line revealed that cis-UCA reduced the production of IL-1β (*P* < 0.001; Fig. 5 B). In an attempt to confirm these findings, we found that cis-UCA decreased by 6.9 times the secretion of IL-1β when compared with UV-B-treated pHCE cells without cis-UCA pretreatment (mean for TNF-α + UV-B: 203.1 pg/mL vs. TNF-α + cis-UCA + UV-B: 29.4 pg/mL, *P* < 0.001; Fig. 5 C). Actually, cis-UCA restored the release of IL-1β back to the baseline level of non-irradiated pHCE cells (TNF-α: 26.5 pg/mL vs. TNF-α + cis-UCA + UV-B: 29.4 pg/mL, *P* > 0.05; Fig. 5 C).

In line with the IL-1β results, cis-UCA reduced the IL-18 secretion by 3.9 times when compared with TNF-α + UV-B-treated cells without cis-UCA (TNF-α + UV-B: 91.5 pg/mL and TNF-α + cis-UCA + UV-B: 23.6 pg/mL, *P* < 0.05; Fig. 5 D). Moreover, cis-UCA restored the production of IL-18 back to the nonirradiated control level (*P* > 0.05;Fig. 5 D). Collectively, these results suggest that cis-UCA was able to inhibit the inflammasome-dependent secretion of IL-1β as well as the inflammasome-independent secretion of IL-18.

In cell viability assays, cis-UCA significantly protected pHCE cells from UV-B-induced cell death (TNF-α + UV-B vs. TNF-α + cis-UCA + UV-B, *P* < 0.001; Fig. 5 E). In line with the cytokine results, LDH release remained at the baseline level if cis-UCA had been provided to the UV-B-irradiated pHCE cells (TNF-α vs. TNF-α + cis-UCA + UV-B, *P* > 0.05; Fig. 5 E).

### cis-UCA has a Therapeutic Effect on the Release of IL-8, IL-6, and LDH in HCE-2 Cells when Applied After the UV-B Exposure

**Figure 6.**
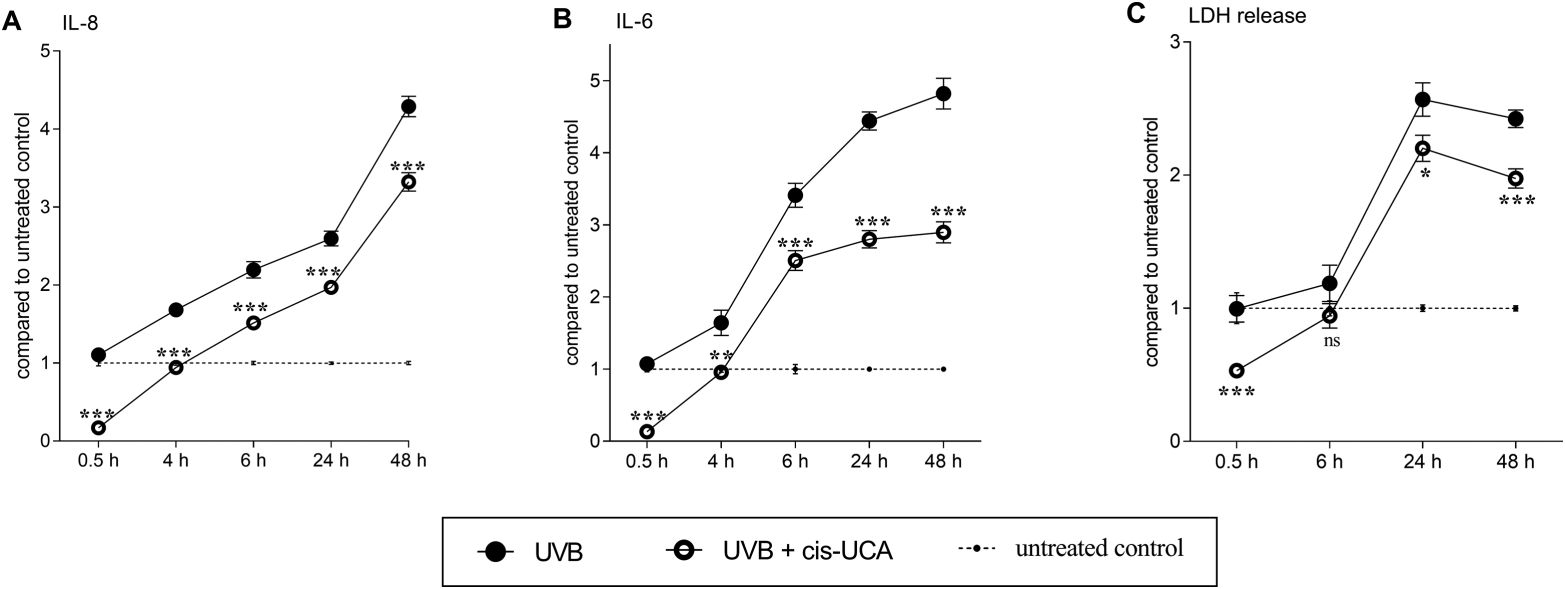
**Therapeutic effect of cis-UCA on the secretion of IL-8, IL-6, and LDH in** **UV-B-exposed HCE-2 cells.** HCE-2 cells were exposed to UV-B (0.2 J/cm^2^) and subsequently treated with cis-UCA (100 µg/mL). Cell culture mediums were collected either 0.5, 4, 6, 24, or 48 hours after the cis-UCA application, and the secretion levels of (A) IL-8 and (B) IL-6 were measured by an ELISA method. (C) Cell membrane integrity was assessed with the LDH assay. (A-C) Results have been converted into the mean of untreated control cells, which is presented in the figure as 1 (dotted line). UV-B (•) and UV-B + cis-UCA (**○**) groups were compared at every time point and the differences were analyzed with the Mann-Whitney *U*-test. Results are represented as mean ± SEM. *P < 0.05, **P < 0.01, ***P < 0.001, ns = not significant.

Finally, we investigated the therapeutic potential of cis-UCA by applying it to HCE-2 cell cultures after the UV-B irradiation. Cis-UCA significantly reduced the secretion of both IL-8 (*P* < 0.001; Fig. 6 A) and IL-6 (*P* < 0.01-0.001; Fig. 6 B) at all five time points (0.5, 4, 6, 24, and 48 hours) when compared with cells exposed only to UV-B. Cis-UCA reduced the secretion of IL-8 and IL-6 even under the basal control level at the 0.5 hours time point (IL-8 from untreated control: 1 vs. UV-B + cis-UCA: 0.2, Fig. 6 A; IL-6 from untreated control: 1 vs. UV-B + cis-UCA: 0.1, Fig. 6 B). According to the cell viability assay assessed with LDH release, already at 6 hours after the UV-B exposure, if cis-UCA was not present, then UV-B slightly increased the rupturing of the cell membrane (Fig. 6 C). At 24 and 48 hours after UV-B-irradiation, the level of LDH was 2.6 and 2.4 times higher when compared with untreated control cells, respectively (Fig. 6 C). Consequently, cis-UCA resulted in 1.2 (*P* < 0.05) and 1.2 (*P* < 0.001) times lower LDH release when this was assessed at 24 and 48 hours after cis-UCA treatment, respectively (Fig. 6 C). Interestingly, even at the 0.5 hour time point, treatment with cis-UCA increased cell viability as compared with the untreated control (untreated control: 1 vs. UV-B + cis-UCA: 0.53, *P* < 0.001; Fig. 6 C).

## Discussion

NLRP3 inflammasomes are intracellular protein complexes, and their activation plays a key role in immune responses.^32^ In the present study, UV-B irradiation was found to induce the NLRP3-mediated activation of caspase-1 and concurrently elevated the levels of IL-1β in the HCE cell line and pHCE cells. This finding is in line with the reports of Feldmeyer et al.^33^ and Hasegawa et al.^34^ that UV-B is able to activate NLRP3 inflammasomes leading to the release of IL-1β from the keratinocytes of human skin.^33^^,^^34^

Although both TNF-α and LPS are able to provide a priming signal in UV-B-stressed HCE-2 cells and can increase the secretion of IL-1β, TNF-α appeared to be a more potent priming agent in this study. In fact, recently it has been claimed that TNF-α is a more selective priming factor for NLRP3 in comparison to LPS.^35^ Additionally, in our corneal cells, UV-B alone resulted in increased IL-1β release in nonprimed HCE cells; even greater cytokine production was dependent on priming, which is a characteristic of the NLRP3 inflammasome.^36^ It has been shown previously that UV-B exposure can trigger the expression of IL-1β mRNA in non-primed HCE cells,^6^ which suggests that UV-B not only activates but also induces the expression of IL-1β. It has been reported that UV-B possesses an ability to activate TNF-R1 receptors on the cell membrane,^37^ explaining the UV-B-dependent expression of pro-IL-1β.

In the present study, intracellular NLRP3 levels were increased in primed pHCE cells but decreased in primed HCE-2 cells after UV-B exposure. Our NLRP3 antibody binds to the N´-terminus of NLRP3 protein (1-150 aa), which is the location of PYD-domain of NLRP3. PYD is a domain that interacts with ASC in the assembly of the inflammasome complex.^38^ Therefore, the changes observed here seem to be dependent on the amount of PYD domain available, irrespective of whether there is a simultaneous activation of the inflammasome. Although UV-B alone increased the production of IL-1β, it did not affect the levels of intracellular NLRP3 in either cultured pHCE or HCE-2 cells. Previously Benko et al.^39^ found that UV-B stimulated the expression of NLRP3 mRNA but the protein concentration did not increase after UV-B irradiation in nonprimed HCE cells, which is in line with the present study. Overall, the levels of intracellular NLRP3 do not reveal whether or not inflammasomes have been activated. The activity of caspase-1 is a more accurate; it became activated in both TNF-α-primed HCE-2 and pHCE cells upon UV-B exposure.

In TNF-α-primed HCE cells, the fate of NLRP3 after inflammasome activation depended on the origin of the cells. Thus, UV-B induced higher secretion of NLRP3 in TNF-α primed HCE-2 cells in comparison to pHCE cells. This may be due to different kinetics in the release of inflammasome particles when comparing a cell line to primary cells. We have recently examined ARPE-19 cells and observed that NLRP3 can be either secreted out of the cells or degraded by autophagy following inflammasome activation.^40^ Additionally, extracellular components of the inflammasome have been detected previously from blood monocyte-derived macrophages, from the serum of patients suffering from cryopyrin-associated periodic syndrome,^41^ and in the bronchoalveolar lavage taken from patients with chronic obstructive pulmonary disease and pneumonia.^42^ Moreover, increased NLRP3 protein levels have been detected from the ocular surface epithelium of patients with non-Sjögren syndrome dry eye,^43^ which could be evidence of the local accumulation of cellular NLRP3 protein in corneal diseases. In the present study, TNF-α alone was also capable of releasing NLRP3 and not simply in pHCE cells followed by UV-B irradiation. It has been reported that TNF-α itself has the capability to induce reactive oxygen species-dependent inflammasome activation and IL-1β production in neuroblastoma cells,^44^ which could support the idea that NLRP3 is secreted out of the cell upon TNF-α-mediated inflammasome activation. According to our data, it appears that TNF-α triggers the secretion of NLRP3 in the absence of an activating signal. This may be the case with the pHCE cells, where NLRP3 was secreted out of the cell in a TNF-α-independent manner on the levels of intracellular NLRP3 or IL-1β. We have recently reported similar findings in Hsp90-inhibited ARPE-19 cells where inactive NLRP3 was secreted out of the cells in addition to being degraded by autophagy.^40^

Apart from IL-1β, the release of another inflammasome-regulated cytokine, IL-18, was studied here from UV-B-stimulated HCE cells. Although priming was needed to enhance the secretion of IL-1β, pro-forms of IL-18 appeared to be constitutively present in HCE cells. In 2001, Burbach et al. were the first to demonstrate the constitutive expression of IL-18 in primary corneal epithelial cells.^14^ Additionally, the same publication revealed that in the corneal epithelial cells, the amounts of secreted and cell-associated IL-18 could be increased by treatment with phorbol 12-myristate 13-acetate, LPS, or double-stranded RNA (poly dI:dC).^14^ Because the IL-18 ELISA kit used in our study measures only the mature form of this cytokine, we concluded that UV-B had induced the secretion of bioactive IL-18. Additionally, we found that IL-18 release was not dependent on the caspase-1 activation, which may indicate that IL-18 does not require NLRP3 inflammasome for its cleavage. Previously, it has been shown that several factors (e.g., epithelial cell-derived metalloproteinase Meprin β,^45^ mast cell derived chymase,^46^ caspase-8,^47^ and NK cell-derived granzyme B^48^) are capable of inducing inflammasome-independent secretion of IL-18. Interestingly, UV-B irradiation has been shown to express granzyme B in human keratinocytes.^49^

Cis-UCA appears to be a promising anti-inflammatory compound because we observed here that cis-UCA reduced the secretion of IL-1β and IL-18 as well as the activity of caspase-1 in HCE cells. Additionally, we demonstrated that cis-UCA possessed therapeutic properties because it decreased the secretion of IL-6 and IL-8 and alleviated cell death even when administered after the UV-B exposure. We have recently confirmed in a clinical phase I study that 0.5% and 2.5% cis-UCA eye drops are well-tolerated after topical administration to the cornea^50^ and there was no evidence of systemic accumulation or systemic or local side effects upon repeated topical administration of 5% cis-UCA in randomized phase I/IIa dermatological trials.^51^ Although the mechanism behind the capacity of cis-UCA to regulate immune responses has been studied widely past 3 decades, it still remains fully unsolved. In our previous studies, cis-UCA was able to prevent IL-8 and IL-6 secretion via the c-Jun/activator protein-1 and JNK/mitogen activated protein kinase pathways.^29^^,^^30^ Instead, Walterscheid et al. have previously reported that the immune suppressive effect of cis-UCA is mediated through the activation of 5-hydroxytryptamine receptor 2 A (5-HTR2A) in mice,^52^ whereas Cloëz-Tayarani and colleagues showed that the stimulation of 5-HT rather increased than decreased the production of IL-1β through 5-HTR2A in LPS-stimulated human PBMCs.^53^ In another study, 5-HT stimulation increased the production of IL-1β but not that of IL-18 in LPS-treated human blood monocytes.^54^ In the study of Dürk et al., IL-1β secretion was mediated via 5-HTR3, 5-HTR4, and 5-HTR7 instead of 5-HTR2A.^54^ In our present study, productions of IL-1β and IL-18 were regulated via distinct signaling pathways but cis-UCA reduced the secretion of both of these cytokines, which along with previous findings propose that IL-1β and IL-18 secretion may not be regulated by 5-HTR2A. Alternatively, cis-UCA has been reported to exert a protodynamic effect in cell cultures, which means that it is capable of transporting protons from a mildly acidic extracellular matrix into the cell.^55^ Because extracellular acidosis is a danger signal for the activation of NLRP3 inflammasome through the K^+^ efflux,^56^ it could be hypothesized that cis-UCA acts to regulate the balance of K^+^ levels between the cytosol and the extracellular space. UV-B is known to trigger K^+^ efflux in human corneal limbal epithelial cells,^57^ and the inhibition of K^+^ efflux is one way to inhibit inflammasome activation and reduce the levels of IL-1β.^58^^,^^59^ Moreover, reactive oxygen species production has been shown to increase the amounts of the pro-form of IL-1β and to promote its maturation via the NLRP3 inflammasome in hyperosmolarity-induced HCE-2 cells.^60^ That could serve as another way to investigate the silencing mechanism of cis-UCA in UV-B-activated inflammasome signaling. The present study provides new insights to study molecular mechanism of cis-UCA, which has now been associated for the first time with the UV-B-induced activation of inflammasomes in human corneal epithelial cells.

Because cis-UCA can inhibit the secretion of IL-1β and IL-18 along with its cytoprotective and therapeutic effects, it may well find extensive applications in several pathological conditions (e.g., pterygium, photokeratitis, CDK, and ocular surface squamous neoplasia where UV-B causes cell damage and inflammation).^3^^,^^4^
